# Solitary fibrous tumors: radiologic features with clinical and histopathologic correlation

**DOI:** 10.3389/fonc.2025.1510059

**Published:** 2025-02-14

**Authors:** Ying Xiao, Jiaer Chen, Wenbin Yang, Hongfei Yan, Ruowei Chen, Yangkang Li

**Affiliations:** ^1^ Department of Radiology, Cancer Hospital of Shantou University Medical College, Shantou, China; ^2^ Department of Pathology, Cancer Hospital of Shantou University Medical College, Shantou, China; ^3^ Department of Medical Imaging, The Second affiliated Hospital of Shantou University Medical College, Shantou, China

**Keywords:** solitary fibrous tumor, radiology, computed tomography, magnetic resonance imaging, pathology

## Abstract

**Objective:**

Solitary fibrous tumor (SFT) is an uncommon mesenchymal neoplasm that exhibits a broad spectrum of biological behaviors. Few studies relative to clinical-pathologic features and radiologic manifestations of SFTs have been reported. This study aimed to correlate the radiologic findings of SFTs with the clinical and histopathologic features.

**Methods:**

The clinical, surgical, and histopathologic records; and CT or MR images in 38 pathologically proved cases of SFTs were retrospectively evaluated.

**Results:**

All tumors were seen as oval (n=18) or irregular (n=20), well-defined (n=36) or ill-defined (n=2) masses with a mean size of 11.0 cm. On CT images, most tumors showed a heterogeneous density on precontrast image, and the mean density of the tumor parenchyma was 40.7 hounsfield units. Intratumoral calcification was seen in 6 tumors. After contrast media administration, most tumors showed moderate to marked enhancement (n=34). Multiple intratumoral vessels were seen in 23 tumors. Collateral feeding vessels were seen in 19 tumors. On MR images, all 6 tumors showed a low signal intensity on T1 weighted images and high signal intensity with separate foci of hypointensity on T2 weighted images, as well as significantly imhomogeneous enhancement after contrast administration.

**Conclusion:**

The presence of a large, well-defined, highly vascular soft tissue tumor with map-like enhancement and prominent collateral feeding vessels should alert the radiologist to the possible diagnosis of SFT. Diagnostic imaging coupled with clinicopathologic analysis allows accurate localization, identification, and resection of SFTs.

## Introduction

Solitary fibrous tumor (SFT) is a rare soft tissue tumor of mesenchymal origin. It was first described as a unique pathological entity of pleura in 1931 by Klemperer and Rabin (1). However, SFTs have been reported in many other extrapleural sites, indicating the mesenchymal rather than mesothelial origin of the tumor (2). The gross and histologic features of SFT may overlap with many other benign and malignant soft tissue tumors, such as leiomyoma, neurofibroma, schwannoma, gastrointestinal stromal tumor, fibrosarcoma, and monophasic synovial sarcoma. With the development of histologic, molecular and genetic techniques, the understanding of SFT has improved and allowed more precise categorization and identification of this type of tumor (3).

Clinically, the tumor is difficult to detect because it usually lacks typical symptoms and signs. Thus, it is often diagnosed in the later stages of the development, when generating mass-occupying effect on the surrounding structures according to their size. At present, radical surgical resection remains the initial management strategy for SFT. Given the variable location of the primary tumor, surgical planning and intervention is highly variable based upon location and involved structures. Preoperative diagnostics need to be systematic to enable the surgeon a precise estimation of the scope of the surgical procedure and tumor operability. Computed tomography (CT) and magnetic resonance imaging (MRI) are preferred initial diagnostic methods for identifying SFTs. They not only aid in establishing the primary diagnosis, but also help in evaluating the extent and blood flow of the tumor and its relationship to adjacent organs. So careful and thoughtful CT and MRI scans in conjunction with the surgical oncologist’s plan result in optimal management of patients.

To our knowledge, quite a few reports concerning the clinicopathological features (2, 4–7) or the imaging characteristics (8–11) of SFT have been published. However, a detailed description of the imaging features of SFT with clinicopathologic correlation in particular was absent in these studies. In the present study, we describe the CT and MRI findings of 38 patients with SFT correlated with clinicopathologic findings, with the aim to be familiar with the imaging appearances of this rare entity and improve the accuracy of preoperative diagnosis.

## Materials and methods

This study was approved by the Ethics Committee of our institution and performed in agreement with the 1964 Declaration of Helsinki. In view of the retrospective study design, the informed consent was waived.

### Patients

Between January 2010 and December 2023, the database of our hospital was reviewed and forty-five patients with pathological proven SFTs were identified. Due to having received treatment or lack of imaging data, seven patients were excluded from the study. Thus, the initial CT or MR images of 38 patients (twenty males and eighteen females) were collected. Regarding cross-sectional imaging, all patients underwent CT examinations (plain scan and biphasic contrast-enhanced scan). 6 patients also underwent MR examinations (precontrast and postcontrast scans). A total of 38 tumors distributing in head and neck (n=2), chest wall (n=1), intrathoracic cavity (n=27), back (n=1), abdominal and pelvic cavity (n=5), buttock (n=1), and distal extremity (n=1) was evaluated.

### CT/MR protocols

CT examinations of 12 patients were performed on a 64-detector-row scanner (Philips Medical Systems, Cleveland, OH, USA), 24 patients were examined using a 16-detector-row scanner (GE Healthcare, Milwaukee, WI, USA), and 2 patient were examined using a GE Lightspeed VCT 64-detector-row scanner (GE Healthcare, Milwaukee, WI, USA). The scanning protocol includes unenhanced and contrast-enhanced CT with arterial and venous phases after intravenous bolus injection of contrast medium (Ultravist 300; Bayer Schering Pharma, Berlin-Wedding, Germany) at a rate of 2.5-3.5 mL/sec and a volume of 60-100 mL. Contiguous axial tomographic images and multiplanar reconstruction (MPR) images were obtained. The section thickness was 5 mm and reconstruction interval was 1.0 mm or 1.25 mm. The volumetric data were transferred to the workstation for further 3D volume rendered (VR) image processing. VR images from the source image datasets were generated by using the commercially available software installed on the workstation. For diagnostic purposes, CT angiographies only containing vascular anatomy were obtained using specific software (Autobone Removal). The software is dedicated to the automatic segmentation of bones from CT angiography data. Moreover, other anatomic structures were removed from the field of interest using volume-punching operations.

MR examinations of 5 patients were performed on a 3.0 T MRI scanner (Discovery MR 750, GE Healthcare, USA). 1 patient was examined using another 3.0 T MRI scanner (Signa HDxt, GE Healthcare, USA). The scanning protocol includes an axial T1-weighted DIXON sequence, a T2-weighted FSE sequence in coronal and axial planes, and a diffusion-weighted imaging sequence. Post-contrast T1 weighted images in three planes were also acquired.

### Image analysis

The CT/MR images were independently evaluated on the workstations by 2 radiologists, who had 12 (W.B.Y.) and 16 (R.W.C.) years of experience in oncological imaging. Their interpretations were arrived at by consensus. The following signs of each lesion were evaluated: tumor size, shape, margin, density/signal, calcification, intratumoral vessels and degree of enhancement. Other accompanying signs, including lymphadenopathy, pleural effusion, ascites and metastasis, were also evaluated.

Specially, the size was measured using the longest diameter as criteria. The shape was depicted as oval or irregular. The margin was described as well-defined or ill-defined. The density/signal was categorized as homogeneous and heterogeneous. Moreover, the tumor density of precontrast and venous phase postcontrast CT images was measured in Hounsfield units (HUs). Circular or elliptical regions of interests (ROI) were marked on the areas which had most enhancements before and after contrast administration. Areas of focal change, such as cystic degeneration, necrosis, calcification, and large vessels, were carefully avoided. To ensure consistency, all measurements were performed three times at different image levels, and the average values were calculated. On CT images, the enhancement degree was described as mild, moderate or severe when the CT value increased by less than 20 HU, 20~40HU or more than 40 HU, respectively. On MR images, the degree of enhancement was subjectively assessed and classified as follows: mild, when the enhancement was similar to that of adjacent muscle; moderate, when the enhancement was higher than that of muscle, but lower than that of blood vessels; and marked, when the enhancement was approaching that of blood vessels. Lymphadenopathy was defined as lymph nodes greater than 10 mm in short axis dimension, abnormal round morphology, or central necrosis. On CT images, the feeding vessels of the tumor were also analyzed using multiplanar reconstruction images and 3D VR images.

After review of the radiologic studies, clinical features, surgical notes and excised specimens were correlated with radiological findings. All statistical data were measurement data, and to be expressed as mean ± standard deviation (x ± s).

## Results

### Clinical findings

There were 20 males and 18 females with a male to female ratio of 1.1:1 and an age of 11 to 83 years. The median age of diagnosis was 54.5 ± 16.2 years. In this cohort, 27 patients (71.1%) were found to have intrathoracic SFTs. For these patients, 10 cases were asymptomatic at presentation and were diagnosed as incidental findings on radiographs or CT images of the chest. The initial symptoms in the other patients were chest pain, non-productive cough, dyspnea, palpitation, or mild peripheral edema. 1 patient concomitantly presented with symptomatic hypoglycemia. For this patient, preoperative routine blood tests were normal with the exception to the serum glucose level at 2.66mmol/L (3.89-6.11 mmol/L), and the hypoglycemia abated postoperatively. 5 tumors originated in the abdominopelvic cavity and the initial symptoms in these patients were due to local pressure effects and included abdominal pain, abdominal distension, nausea and vomiting, or lower urinary tract symptoms. For the other 6 patients, the initial symptom was a palpable painless mass with slow growth. Clinical findings of all patients summarized in **Table 1**.

**Table 1:** Clinical findings of 38 patients with solitary fibrous tumor.

Location	Patient (case)	Age (y)	Sex		Symptom
			Male (n)	Female (n)	
Intrathoracic cavity	27	54.5 ± 16.2	14	13	asymptomatic (n=10), chest pain, non-productive cough, dyspnea, palpitation, peripheral edema, symptomatic hypoglycemia
Abdominal cavity	1		0	1	abdominal pain, abdominal distension, nausea and vomiting, frequent micturition, urgent micturition, dysuria, urinary hesitancy, urinary incontinence
Retroperitoneal space	2		2	0	
Pelvic cavity	2		2	0	
Head and neck	2		2	0	a slow growing painless mass
Chest wall	1		0	1	
Back	1		0	1	
Buttock	1		0	1	
Distal extremity	1		0	1	
**Total**	38		20	18	

### Radiological findings

**Figure 1:**
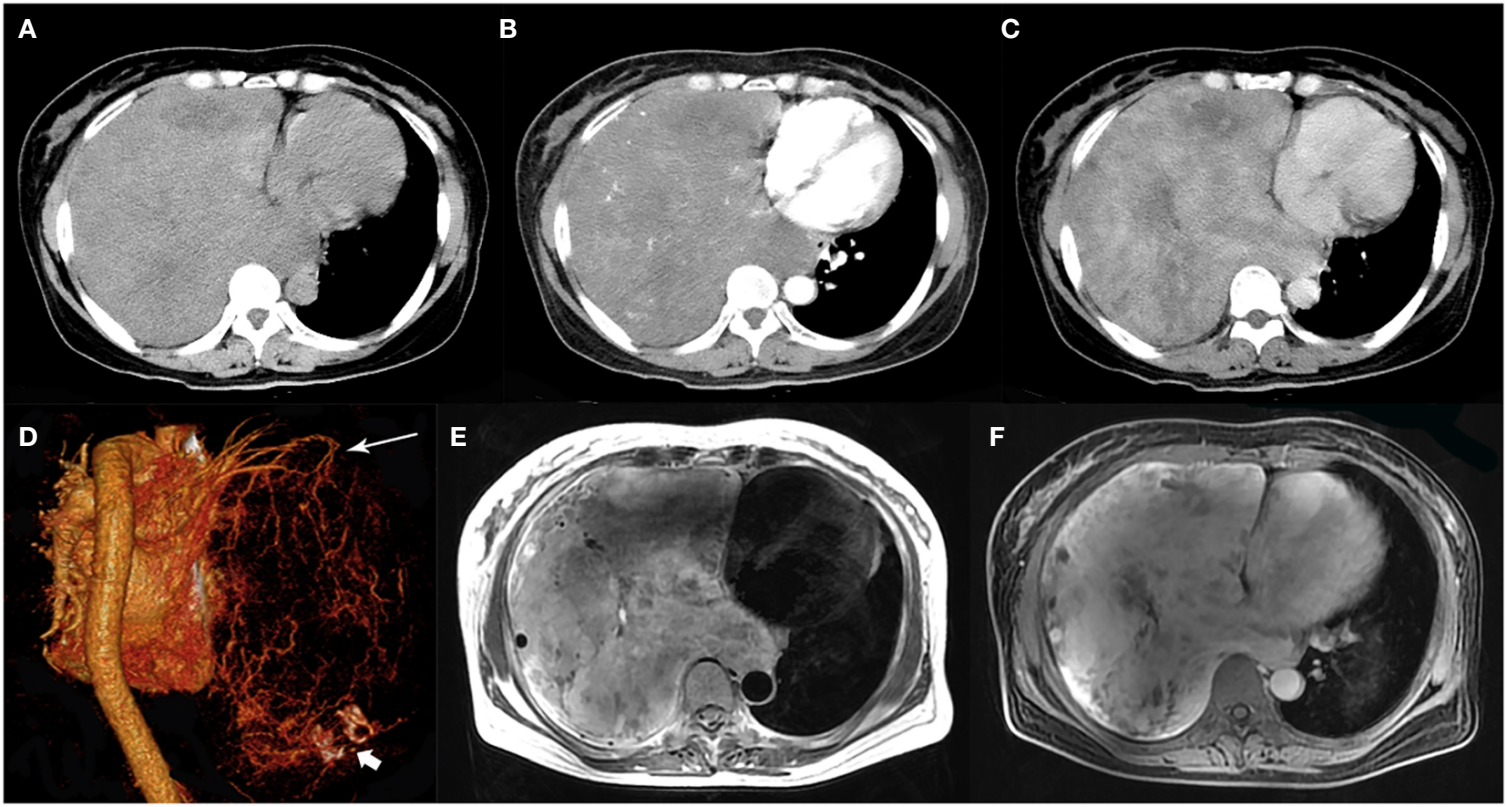
A 51-year-old woman with SFTP. **(A)** Precontrast CT scan shows a huge mass with relatively high density in the right thoracic cavity. **(B)** Numerous enlarged intratumoral vessels are seen in the arterial phase on post-contrast CT image. **(C)** Heterogeneous marked enhancement is detected in the venous phase. **(D)** Collateral feeding arteries arising from the right upper pulmonary artery are detected on 3D VR image (white arrow). Intratumoral calcification is also detected (white arrow head). **(E)** A black-and-white mixed pattern and multiple flow voids are seen on T2-weighted image. **(F)** Heterogeneous moderate to marked enhancement are seen on postcontrast MR image.

**Figure 2:**
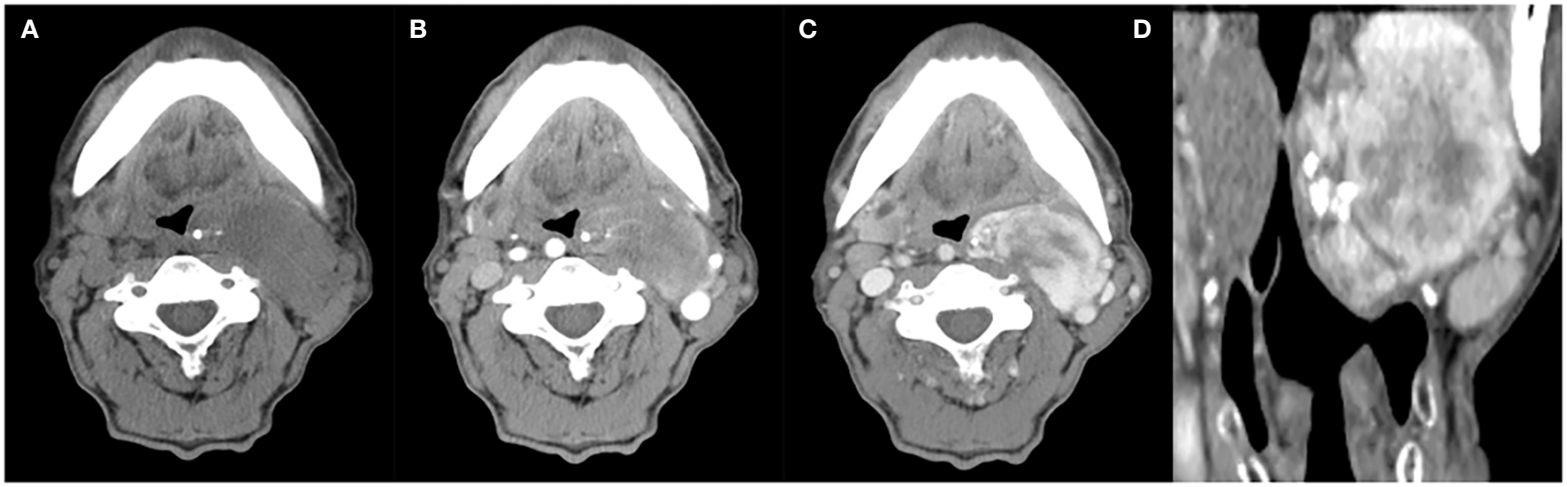
A 63-year-old man with SFT. **(A)** Precontrast CT scan shows an oval mass with calcification in the left parapharyngeal space. **(B)** Several enlarged intratumoral vessels are seen in the arterial phase on post-contrast CT image. **(C, D)** Heterogeneous marked enhancement is detected in the venous phase.

**Figure 3:**
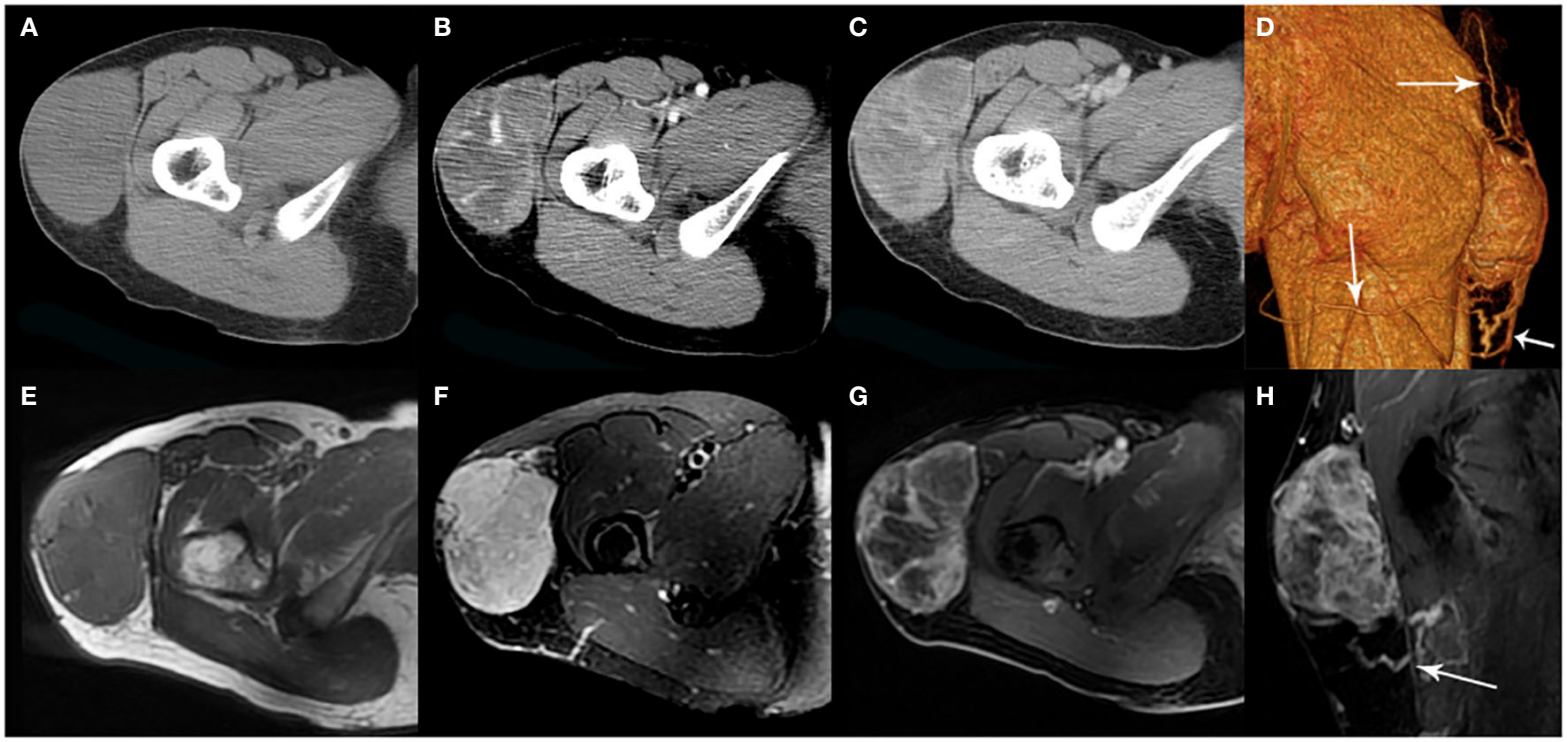
A 35-year-old woman with SFT. **(A)** Precontrast CT scan shows a mass in the proximal right thigh. **(B)** Multiple enlarged intratumoral vessels are seen in the arterial phase on post-contrast CT image. **(C)** Heterogeneous marked enhancement is detected in the venous phase. **(D)** Collateral feeding arteries are detected on 3D VR image (white arrows). **(E)** The tumor shows low intensity on T1-weighted image. **(F)** Areas of hypointensity and multiple flow voids were seen on T2-weighted image. **(G, H)** Heterogeneous moderate to marked enhancement and prominent collateral feeding artery (white arrow) are seen on postcontrast MR images.

**Figure 4:**
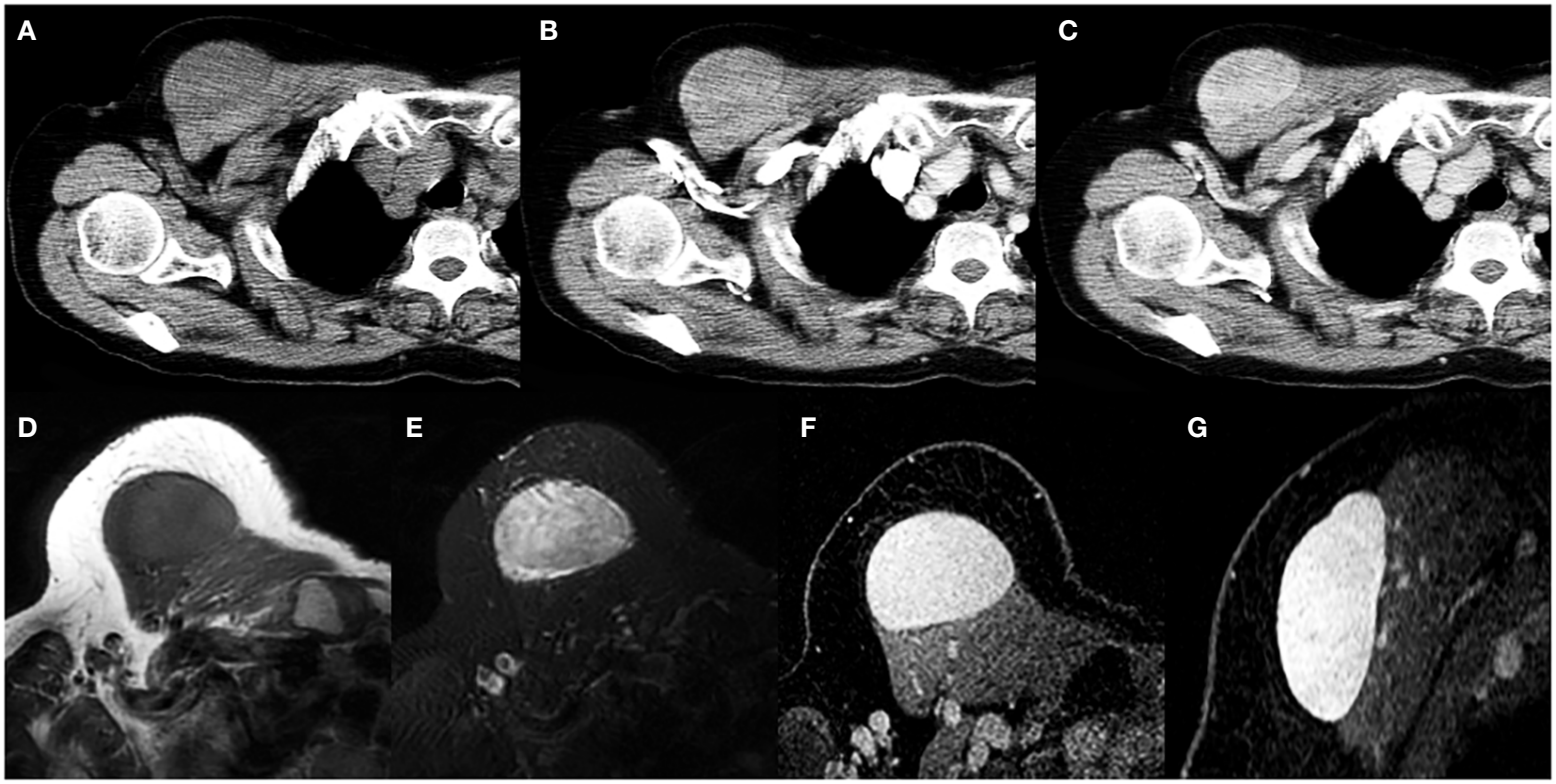
A 69-year-old woman with SFT. **(A)** Precontrast CT scan shows an oval mass in the anterior chest wall. **(B)** No obvious intratumoral vessels are seen in the arterial phase on post-contrast CT image. **(C)** Heterogeneous marked enhancement is detected in the venous phase. **(D)** The tumor shows low intensity on T1-weighted image. **(E)** A black-and-white mixed pattern is seen on T2-weighted image. **(F, G)** Heterogeneous moderate to marked enhancement are seen on postcontrast MR images.

**Figure 5:**
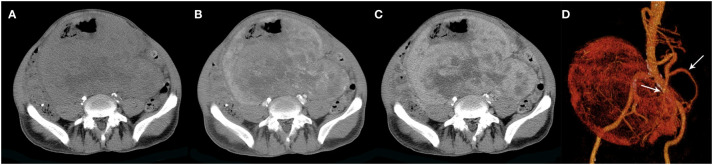
A 60-year-old man with SFT. **(A)** Precontrast CT scan shows a huge irregular mass with areas of necrosis in the pelvic cavity. **(B)** Numerous intratumoral vessels are seen in the arterial phase on post-contrast CT image. **(C)** Heterogeneous marked enhancement is detected in the venous phase. **(D)** Collateral feeding arteries arising from the inferior mesenteric artery are detected on 3D VR image (white arrows).

The CT images of all 38 tumors were analyzed. The size of the tumors ranged from 0.8 to 27.5 cm (mean, 11.0 ± 6.6 cm). The shape was seen as oval (n=18) or irregular (n=20). 36 tumors showed a well-defined margin, and 2 tumors showed an ill-defined margin. Dense central or punctate calcification was seen in 6 tumors. On precontrast CT images, the density was homogeneous (n=7) or heterogeneous (n=31). The mean density of the solid component of all tumors was 40.7 ± 6.4HU with a range of 27-53HU (**Figures 1**–**5A**). After contrast medium administration, abundant intratumoral vessels were seen in 23 cases in the arterial phase (**Figures 1**–**3B**, **5B**). The remaining 15 cases showed no obvious intratumoral vessels (**Figure 4B**). In the venous phase, the mean density of the solid component of all tumors was 81.3 ± 24.6HU with a range of 45-186HU, and there were marked enhancement in 18 (**Figures 1**–**5C**, **2D**), moderate enhancement in 16, or mild enhancement in 4. Collateral feeding vessels of the tumor were identified in 19 cases. The arteries branched into a spiderlike series of vessels on the surface of the tumor (**Figures 1D**, **3D**, **5D**).

On MR imaging, all 6 masses exhibited a low T1 signal intensity and a high T2 signal intensity with separate foci of hypointensity (**Figures 1E**, **3E, F**, **4D, E**). Multiple flow voids on T2-weighted images were seen in 4 masses (**Figures 1E**, **3F**). Heterogeneous moderate to marked enhancement after contrast administration was seen in 5 masses (**Figures 1F**, **3G, H**, **4F, G**).

No patient had local or distant lymph node involvement. A small amount of pleural effusion was seen in 6 cases and significant pleural effusion was seen in 1 case. No ascites was detected. Pleural or hepatic metastasis occurred in 2 cases with malignancy.

CT and MRI findings of SFTs are summarized in **Table 2** and **Table 3**.

**Table 2:** CT findings of 38 solitary fibrous tumors.

CT Features	Tumors (n)	Percentage (%)
Diameter (max, cm)	11.0 ± 6.6	
Precontrast density (HU)	40.7 ± 6.4	
Homogeneous	7	18
Heterogeneous	31	82
Shape		
Oval	18	47
Irregular	20	53
Margin		
Well-defined	36	95
Ill-defined	2	5
Calcification		
Present	6	16
Absent	32	84
Intratumoral vessels		
Present	23	61
Absent	15	39
Enhancement degree		
Mild	4	11
Moderate	16	42
Marked	18	47
Collateral feeding vessel		
Present	19	50
Absent	19	50
Pleural effusion/ascites		
Present	7	18
Absent	31	82
Metastasis		
Present	3	8
Absent	35	92

**Table 3:** Imaging characteristics of solitary fibrous tumor.

Imaging Modality	Findings
CT	Well-defined margin Relatively high density on precontrast images Multiple intratumoral vessels in the arterial phase on postcontrast images Obvious heterogeneous enhancement in the venous phase on postcontrast images Prominent collateral feeding vessels
MRI	High T2 signal intensity with separate foci of hypointensity Multiple flow voids on T2-weighted images Obvious heterogeneous enhancement on postcontrast images

### Histopathological findings

**Figure 6:**
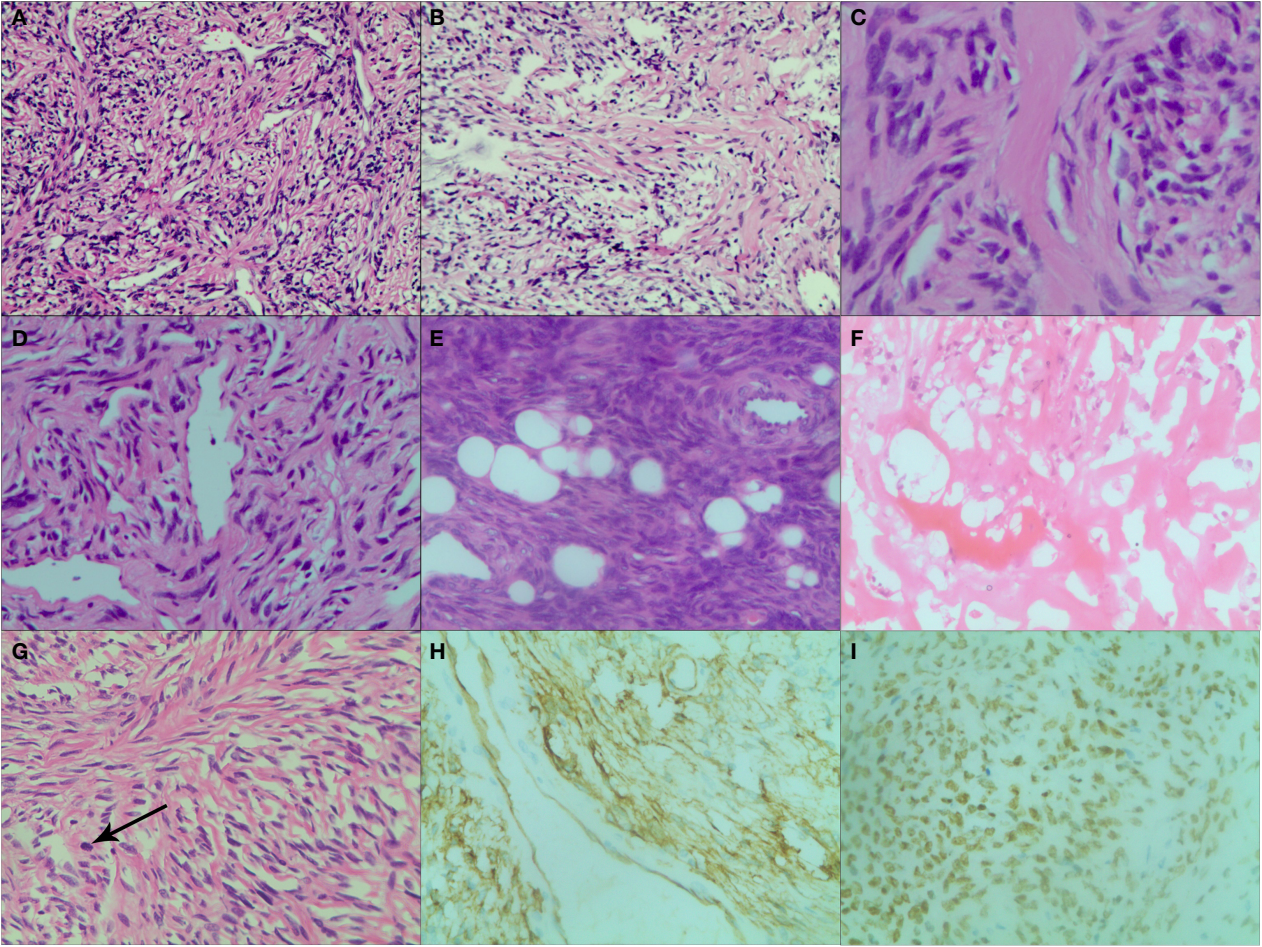
Various histopathological patterns in SFT. **(A)** Numerous spindle-ovoid cells in a herringbone or fascicular pattern with multiple irregular staghorn-type, variably dilated and branching thin-walled vessels (HE ×200). **(B)** Numerous spindle-ovoid cells in a patternless fashion with variably dense, intervening fibrous stroma (HE ×200). **(C)** Hypocellular areas with prevailing fibrous stroma (HE ×400). **(D)** Hemangiopericytoma-like vasculature pattern (HE ×200). **(E)** Extensive lipomatous differentiation with spindle-ovoid cells (HE ×200). **(F)** Necrosis and hemorrhagic zone (HE ×200). **(G)** Spindle cells in a storiform pattern with scattered mitoses (black arrow) (HE ×200). **(H)** Strong and diffuse cytoplasmic CD34 immunoreactivity (×200). **(I)** Strong and diffuse nuclear STAT6 immunoreaction (×200).

35 cases underwent needle biopsy, and 34 cases underwent surgery. Intraoperatively, all tumors were completely removed. Especially, for 27 cases with intrathoracic SFTs, 25 cases underwent surgical resection and were proved pleural SFTs (SFTPs). Macroscopically, the resected tumors demonstrated as oval or lobular shape, well-circumscribed margin, smooth and encapsulated external surface with extensive vessels and gray-to-red or gray-to-white on cross-section. Microscopic examination revealed a proliferation of spindle-shaped cells in a patternless or fascicular or partial storiform fashion with thin-walled branching vessels and dense bands of collagen. A hemangiopericytoma-like vascular pattern and lipomatous (fatforming) differentiation may also occur. Moreover, areas of hemorrhage, necrosis, cystoid areas or mucoid material were seen in large tumors. Malignant SFT was diagnosed if >4 mitoses/10 HPF were present, which was present in 15 cases. In immunohistochemical examinations the following results were obtained: positive results for CD34 in 34 cases (89%), for CD99 in 23 cases (61%), for BCL-2 in 20 cases (53%), for Vimentin in 24 cases (63%), for Ki-67 in 10 cases (26%). Especially, the Ki-67 index was analyzed as the percentage of positive cells with nuclear staining in average of five high power field. The score was considered to be positive if the expression was equal to or greater than 10% and negative if the expression was less than 10%. STAT-6 staining was evaluated in 12 tumors and the positive rate was 100%. The histopathological findings of SFTs are showed in **Figure 6**.

## Discussion

SFT is a kind of rare soft-tissue tumor of mesenchymal origin. Because of the ubiquitous nature of mesenchymal tissue, SFT has the potential of being found in all organs. In fact, descriptions of SFTs show that they typically occur in the thorax but may also arise elsewhere, such as the head, neck, abdomen, pelvis and extremities. In this cohort, only 11 tumors (11/38, 29%) originated outside the thoracic cavity, in agreement with the prior report (12). In the present study, SFTs were found to have a peak incidence in the fifth decade of life, and affect both sexes equally. This distribution pattern is also consistent with the previous study (6). Asymptomatic SFTs were found in 26.3% of patients and most clinical symptoms caused by tumors were non-specific. Most tumors presented as slow growing painless masses with large size (mean maximal diameter, 11.0 cm) and well-defined border. The above clinical findings may be associated with the origin of this kind of tumor. SFTs originate in mesenchymal cells not in the epithelium. They tend to grow in an expansive centripetal fashion and cause pressure on peripheral structures when the tumor volume is large enough. Neighboring organs are usually displaced, rather than invaded.

Pathologically, microscopic features of SFTs were various, even within a single tumor, including areas of spindle cells with patternless fashion, herringbone or fascicular pattern, storiform pattern and hemangiopericytoma-like pattern, or hypocellular areas with prevailing fibrous stroma. Extensive lipomatous differentiation was also detected. Areas of hemorrhage, necrosis, cystoid areas or mucoid material were seen in large tumors. The above pathological findings were consistent with literature reports (13, 14). Because of the fascicular proliferation and the prominent branching hemangiopericytoma like vasculature, SFT may mimic some other soft tissue tumors histologically. In the past, the combination of the classic morphology and immunohistochemical positivity of CD34, CD99 and BCL2 was mandatory for diagnosis of SFT. However, CD34 is not a specific immunomarker of SFT and it can be negative in about 10% of SFTs, furthermore, CD99 and BCL2 are usually expressed in many other neoplasms (15). Strong, diffuse nuclear staining for STAT6 has been proven to be highly specific for SFT, and allows pathologists to confidentially make a diagnosis of SFT even on small preoperative needle biopsies (16). In our study, although STAT6 staining was only evaluated in 12 tumors, the positive rate was 100%.

In the present study, small SFTs appeared as homogeneous masses and large tumors depicted obviously heterogeneous pattern on precontrast images. In our opinion, large tumor size is a reason of tumor heterogeneity. Because it may outgrow tumor blood supply and cause intratumoral hemorrhage, necrosis or cystic change. Moreover, myxoid degeneration also contributes to tumor heterogeneity. The above intratumoral pathological changes were also confirmed on cut sections and microscopic examinations. However, the substantial components within the tumor were found to be relatively hyperdense on precontrast CT images or low signal on T2-weighted MR images, regardless of the tumor size. The imaging feature of SFTs reflects their characteristic histopathologic appearances. As presented in our report, pathological results revealed that the tumor contained a mixture of areas of hypercellular regions, generous collagenous hypocellular regions and multifocal myxoid degeneration, which correspond to a relatively high density on precontrast CT images or a black-and-white mixed pattern on MR images. The low signal on T2WI was considered to be an important MR imaging feature of SFTs (10).

A pattern of moderate to marked enhancement was found in most tumors in this study. This sign was more common in larger tumors and was depicted more clearly in the venous phase after contrast administration on CT images. It means that delayed enhancement occurs in some regions of the tumor. The term “geographic enhancement” was used to depict this imaging finding (17, 18). Good enhancement reveals the hypervascularity of the tumor parenchyma. Hypervascularity usually leads to intratumoral bleeding and may be another reason of tumor heterogeneity on precontrast images.

On T2WI and post-contrast CT images, there were abundant vessels are detected in the tumor, which also suggests the hypervascularity of the tumor. Different with the previous sign, it was described more clearly in the arterial phase after contrast administration on CT images. This means that the blood vessels inside the tumor may originate from the feeding arteries of the tumor. Moreover, in the venous phase, significant enhancement of the tumor parenchyma may also cover up this sign. Cardinale et al. reported massive intratumoral vessels in approximately one fourth of their cases (17). Wignall et al. also reported that numerous dilated intratumoral vessels and avid contrast enhancement were observed in 22 of the 34 tumors on post-contrast CT images (19). In our series, this sign is detected in 23 SFTs (61%) and can be considered as a valuable imaging feature in diagnosing SFT.

The other distinctive imaging feature of SFT is the presence of prominent collateral feeding vessels, which was seen in 19 cases (50%). We found that the SFTs had this sign usually present as a larger mass with the size ranging from 6.0 to 27.5cm (mean, 14.7cm). In our experience, although it is not specific, this sign is helpful when present and can aid the radiologist in narrowing the differential diagnosis. The vascular nature of the tumors and the presence of large collateral feeding vessels usually made surgical removal technically difficult. Thus, besides helping the radiologist to diagnose SFT, another potential benefit of this imaging feature is in planning for therapy (20). Radiologists should caution surgeons about the possibility of massive bleeding occurring during biopsy or resection and should suggest vascular embolization or ligation to minimize the risk of operative hemorrhage.

Calcifications, pleural effusions and ascites are uncommon findings in SFT. In our study, calcification was only seen in 6 cases (16%). A small amount of pleural effusion was seen in 6 cases of 29 intrathoracic SFTs. No ascites was detected in all 5 abdominopelvic SFTs. The findings are similar to those in previous reports (12, 21). It means that the presence or absence of calcification, hydrothorax or ascites is not a helpful distinguishing feature on CT or MR imaging.

It is often difficult to differentiate between benign and malignant forms of SFT by radiological imaging (12, 22–24). Radiological features which are suggestive of malignancy include lesions larger than 10cm, infiltrative margins with surrounding tissue, large pleural effusions or ascites, and metastasis. In the current study, 15 malignant SFTs were reported. They usually show as a huge mass with the size ranging from 4.0 to 27.5cm (mean, 13.7cm). However, an ill-defined margin, severe hydrothorax and metastasis were only seen in 2, 1 and 2 cases, respectively. The pathologic criteria of malignant SFTs vary from study to study (23–27), but malignant lesions are hypercellular with at least focal moderate to severe nuclear atypia. They often have necrosis or hemorrhagic zones, exhibit stromal or vascular invasion, and have high mitotic rate (≥4 mitoses per 10 high-power fields).

In this study, we also reported 1 patient with pleural SFT who presented with symptomatic hypoglycemia. The tumor appeared as a massive mass in the lower left thoracic cavity. Hypoglycemia is a rare paraneoplastic symptom caused by SFT and has been found in 4-5% of patients, so called non-islet cell tumor hypoglycemia (NICTH) (23). This hypoglycemic response is due to tumor overproduction of a higher molecular weight form of IGF-II (28). Surgical resection of the tumor is curative and is the gold standard treatment. Base on reviewing the literature, we found that hypoglycemia was exclusively observed in large pleural SFTs, and was rarely documented in small pleural SFTs or extrapleural SFTs (29–32). In our opinion, when the imaging features of a large pleural based tumor are atypical, the presentation of hypoglycemia may be a clue to diagnose SFT.

There are several limitations in our study. Firstly, this was a retrospective study. Secondly, the study was performed on a small series of cases, especially for extrathoracic SFTs. Thirdly, different CT and MR equipments were used, and MRI was only performed on a few patients. Fourthly, a detailed study was absent to be correlated the imaging findings with patients’ prognosis. Additional study should be taken into consideration when analyzing radiologic and clinical-pathologic features along with prognostic factors, which will help to more comprehensively characterize this kind of tumor.

## Conclusions

SFT is a rare mesenchymal tumor occurring at any age and any location, with incidence peak in the 5th decade of life, affecting both sexes equally and more often associated with the pleura. Clinical symptoms of patients with SFTs are non-specific and about one-third of cases are incidentally discovered without any symptoms. Symptomatic hypoglycemia is a rare but distinctive clinical feature of patients with large pleural SFT. CT and MRI are important modalities to evaluate the location, size, internal structures, blood flow of the tumor and its relationship to adjacent organs preoperatively. The diagnosis is based on histopathological examinations, which also reflect the characteristic imaging findings of this kind of tumor.

## Data Availability

The original contributions presented in the study are included in the article/supplementary material. Further inquiries can be directed to the corresponding author.
